# Iron-Catalyzed Stereospecific Heterocycle *N*-Glycosylation with Glycal Epoxides

**DOI:** 10.21203/rs.3.rs-8650471/v1

**Published:** 2026-01-21

**Authors:** Xiao-Wen Zhang, Dakang Zhang, Zixiang Jiang, Hao Xu

**Affiliations:** Brandeis University; Brandeis University; Brandeis University; Brandeis University

## Abstract

Stereospecific *N*-glycosylation of heterocycles with glycal epoxides could readily provide valuable building blocks for drug discovery, but heterocycle *N*-glycosylation with a pyranose-based glycal epoxide is still difficult using existing methods. We report herein an iron-catalyzed, stereospecific heterocycle *N*-glycosylation method for these glycal epoxides in high yields and with low catalyst loadings. This method is functional-group tolerant and effective for a wide variety of functionalized, complex glycal epoxides and heterocycles.

Heterocycle *N*-glycosylation plays an important role in the discovery of nucleoside-based therapeutics for infectious diseases (Figure 1A). The pioneering Vorbrüggen reaction^1^ (reaction of a silylated pyrimidine with a glycosyl acetate in the presence of a Lewis acid) is still practiced today and can be scaled up to the multi-kilogram scale for the synthesis of a complex nucleoside analogue.^2^ However, development of effective and functional group-tolerant catalytic methods for heterocycle *N*-glycosylation remains a challenge.^3^ The Lewis-basic heterocyclic nitrogen atoms often deactivate Lewis acid catalysts and promoters so that the glycosylation reaction requires forcing conditions. Additionally, the basic reaction medium that facilitates most C–N bond forming reactions is less compatible with functional groups presented in commonly used glycosyl donors.

These challenges inspired the development of an array of valuable heterocycle *N*-glycosylation methods,^4–26^ but a mechanistically distinct approach could capitalize on the stereospecific *N*-glycosylation with glycal epoxides (Figure 1B). It is known that a furanose-based glycal epoxide can readily glycosylate a silylated or a deprotonated heterocycle in the absence of a catalyst or a promoter,^27–32^ but stereospecific *N*-glycosylation with a pyranose-based glycal epoxide is still difficult. The existing methods require electron-rich, persilylated and perbenzylated glycosyl donors and even these have limitations (Figure 1B).^32–33^

A stoichiometric ZnCl_2_-mediated method afforded an *N*-glycosylated thymine in modest yield.^32^ An *N*-glycosylation promoted by a sub-stoichiometric amount of TMSOTf had to be operated at elevated temperatures leading to decreased *dr* (Figure 1B).^33^ Thus, a generally applicable and functional group-tolerant, heterocycle *N*-glycosylation method with glycal epoxides has yet to be developed. We have recently discovered the iron-catalyzed highly stereospecific glycosylation of hindered secondary sugar acceptors with glycal epoxides.^34^ Building upon this discovery, we report here an iron-catalyzed stereospecific heterocycle *N*-glycosylation method that is functional group-tolerant and effective for a wide variety of complex glycal epoxides (Figure 1C).

Electron deficient glucuronic acid-based glycosyl donors are less reactive and therefore difficult to activate.^35–38^ Only limited catalytic methods are effective in promoting stereospecific glycosylation with glucuronic ester epoxides.^34–35^ Therefore, we selected glucuronal **2** as a model substrate for reaction discovery (Figure 2). Epoxidation of glucuronal **2** in a biphasic reaction medium with Oxone^^®^39^ quantitatively afforded the corresponding glucuronic ester a-epoxide (Figure S1, *dr* >20:1, ^3^*J*_*H1-H2*_ = 2.6 Hz),^40^ which was azeotropically dried and used directly. Bis-silylation of uracil (**3**) with *N*,*O*-bis(trimethylsilyl)trifluoroacetamide (BSTFA)^41^ followed by solvent exchange (MeCN^®^CH_2_Cl_2_) quantitatively generated the activated glycosyl acceptor. Extensive exploration of catalysts and other reaction parameters revealed that the readily available, hemin-derived iron catalyst **1a** (5 mol %) used for stereospecific glycosylation with hindered sugar acceptors^34^ is optimal in promoting this *N*-glycosylation at 0 °C in 2 h to afford **4** (91% yield, *dr* >20:1). Further experiments suggested that both the iron catalyst **1a** and bis-silylation of the glycosyl acceptor **3** are crucial for the effective glycosylation (Figure 2. entries 1–2). Replacement of catalyst **1a** with either AgOTf, TMSOTf, Fe(OTf)_2_, or iron catalyst **1b**^42–43^ resulted in significantly lower reactivity (<5% conversion in 2 h in Table S1). The glycosylation in prolonged time (24 h) afforded **4** in 13–21% yields with a variety of byproducts (entries 3–6 of Figure 2). We observed that this glycosylation can be carried out in acetonitrile used for pyrimidine bis-silylation without solvent exchange, albeit with a slower rate (entry 7) and that the *N*-glycosylation product **4** can still be obtained in 90% yield with a low catalyst loading (2 mol %) by increasing the reaction time to 6 h (entry 8).

With the optimal catalyst **1a** confirmed, we explored a variety of glycals and heterocycles to determine the generality of this method (Figures 3 and 4). An *N*-glycosylated uronic ester is a valuable building block for the synthesis of Ningnanmycin-type antibiotics and Gougerotin.^44^ Therefore, we are particularly interested in heterocycle *N*-glycosylation with glycal epoxides derived from highly electron-deficient glucuronal **S2** and galactoronal **S4** (Figure 2). The epoxides obtained from these two glycals can be smoothly converted to the *N*-glycosylated uracils in good yields (products **4**–**6**, *dr* >20:1). Next, we examined an array of electronically differentiated glucals and galactals, as well as a 6-deoxy glucal and a xylal: all of them are excellent substrates and the corresponding glycal epoxides can readily *N*-glycosylate a bis-silylated uracil in excellent yield (products **7**–**13**,*dr* >20:1). To probe for the functional-group compatibility and synthetic utility of this method, we subsequently evaluated a range of readily available, disaccharide-based glycosyl donors,^34^ including the epoxides derived from glucosamine (GlcN)-a-1,6-glucose (Glu), GlcN-a-1,4-glucuronic acid (GlcA), GlcN-a-1,3-Glu, GlcN-b-1,3-Glu, as well as those from maltose and lactose. Using iron catalyst **1a**, the *N*-glycosylation with all of these donors afforded single diastereomeric products in high yield (products **14**–**19**,*dr* >20:1). It is also worth mentioning the bis-silylated thymine and *N*-benzoyl cytosine are both compatible with this method affording *N*-glycosylated thymines and cytosines in good yields (products **20**–**26**,*dr* >20:1).

Interestingly, heterocycles with multiple Lewis-basic nitrogen atoms can directly participate in this iron-catalyzed stereospecific *N*-glycosylation without BSTFA activation (Figure 4). 1,4-Dioxane is a necessary co-solvent for heterocycles that have low solubility in CH_2_Cl_2_, and longer reaction time is often needed for full conversion. Ribosylated benzimidazoles are promising inhibitors of human cytomegalovirus (HCMV),^45^ so we first evaluated benzimidazole *N*-glycosylation with an array of functionalized, pyranose-based glycal epoxides. All of these glycosylations afford the desired products in good yield (products **27**–**33**, *dr* >20:1). 1,2,4-Triazoles are synthetically valuable because of their relevance to antiviral medicine Ribavirin.^46^ We observed that the catalytic *N*-glycosylation occurs regioselectively at the triazole N1 position in decent yields (corresponding products **34**–**36**, *dr* >20:1, see Supporting Information for details). Furthermore, we explored the *N*-glycosylation of an indazole and a tetrazole: both of the regioselectively *N*-glycosylated heterocycles were isolated in excellent yields (corresponding products **37**–**42**, *dr* >20:1, see Supporting Information for details).

Most known purine *N*-glycosylation methods predominantly afford the *N*9-glycosylated adenines. The initially formed *N*3-glycosylated adenine undergoes an irreversible *N*3 to *N*9 transglycosylation, presumably by formation of the 3,9-*N*,*N*’-diglycosylated adenine and cleavage of the *N*3 glycoside.^47–48^ However, the iron-catalyzed glycosylation of bis-(*N*6-Boc)-protected adenine **S24** affords both the *N*9-(**43a**–**45a**) and the *N*3-glycosylated adenines (**43b**–**45b**) in excellent combined yield. Notably, **43a** and **43b** do not interconvert under the reaction conditions (Figure S7) and they are readily separable by column chromatography, providing an expedient way for the synthesis of 3-isoadenosine analogs. Interestingly, the iron-catalyzed *N*-glycosylation of a protected guanine **S27** exclusively generated the *N*9-glycosylated guanines (**46** and **47**).

In conclusion, we have developed an iron-catalyzed highly stereospecific heterocycle *N*-glycosylation method with pyranose-based glycal epoxides. This method is effective for a wide variety of glycals and heterocycles and it is compatible with an array of functional groups often used in complex-glycan synthesis. Our current effort focuses on applications of this method in rapid synthesis of small-molecule therapeutics.

## Supplementary Files

This is a list of supplementary files associated with this preprint. Click to download.

- XuIronCatalyzedHeterocycleNGlycosylationSIDecember29.pdf
- Screenshot20260121205303.png

Supporting Information

Experimental procedure, characterization data for all new compounds and selected NMR spectra. The Supporting Information is available free of charge on the ACS Publications website.

## Figures and Tables

**Figure 1:**
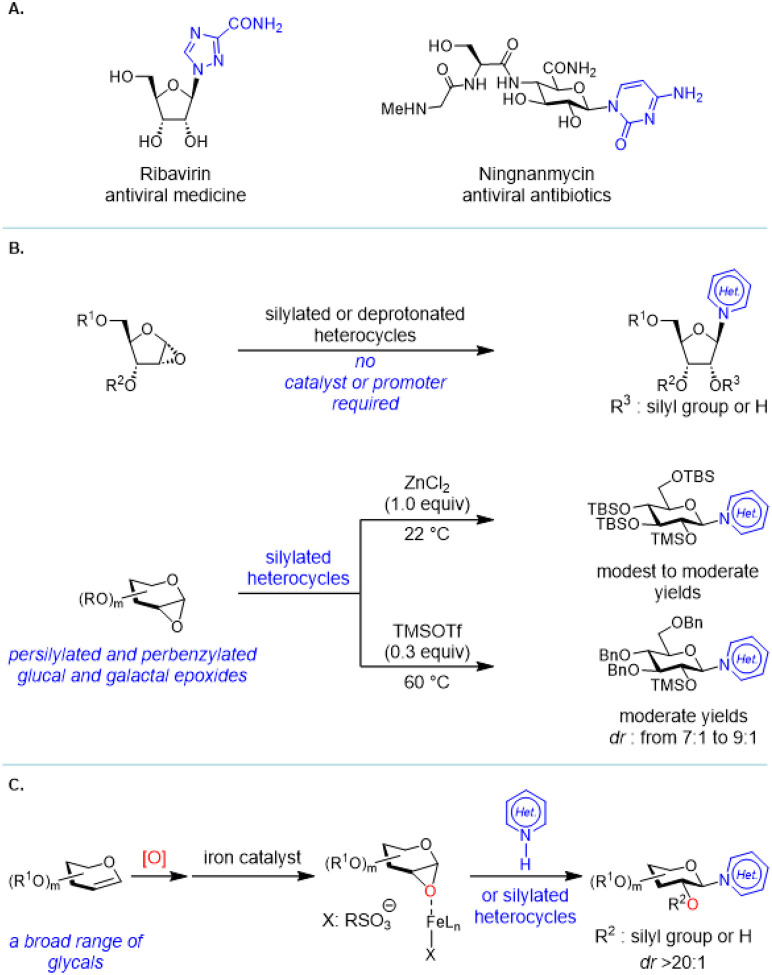
(A) Biologically active *N*-glycosylated heterocycles: Ribavirin and Ningnanmycin. (B) Existing heterocycle *N*-glycosylation methods with glycal epoxides. (C) This research: iron-catalyzed stereospecific heterocycle *N*-glycosylation with glycal epoxides.

**Figure 2:**
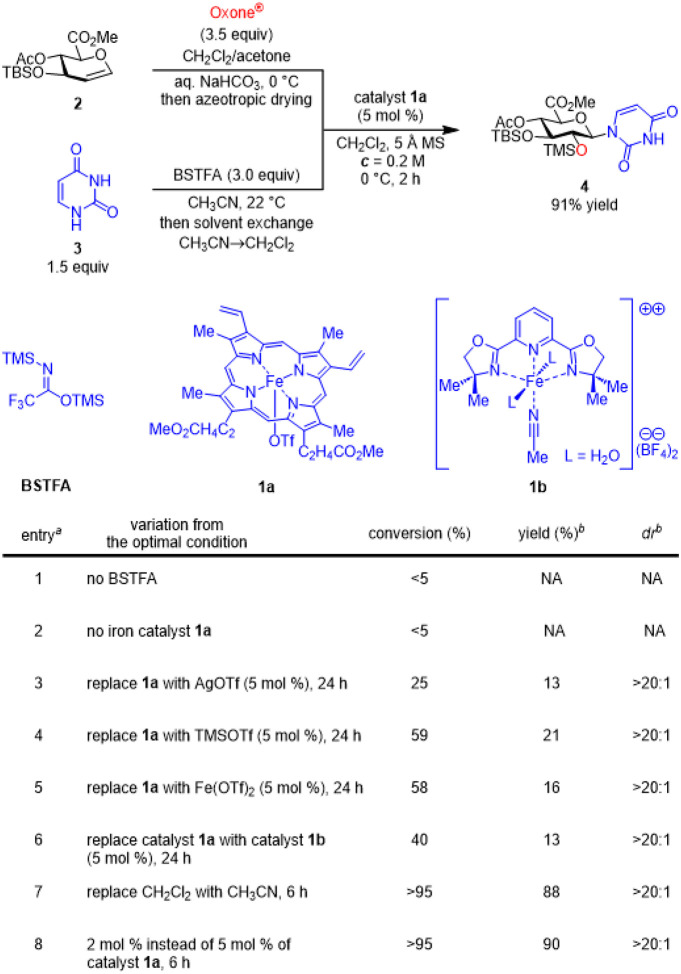
Catalyst discovery for the iron-catalyzed stereospecific heterocycle *N*-glycosylation with glycal epoxides. ^a^Epoxidation was carried out in a biphasic reaction medium with Oxone^®^ and acetone. The glycal epoxide was dried azeotropically with toluene, assayed by ^1^H NMR, and then directly used. The glycosylation was carried out at 0 °C in CH_2_Cl_2_. The reaction was quenched by methanol and imidazole for conversion measurement. ^b^Isolated yield; *dr* was determined by ^1^H NMR analysis. Iron(III) porphyrin catalyst **1a** was formed in situ from the corresponding iron porphyrin chloride (6 mol %) and AgOTf (5 mol %).

**Figure 3:**
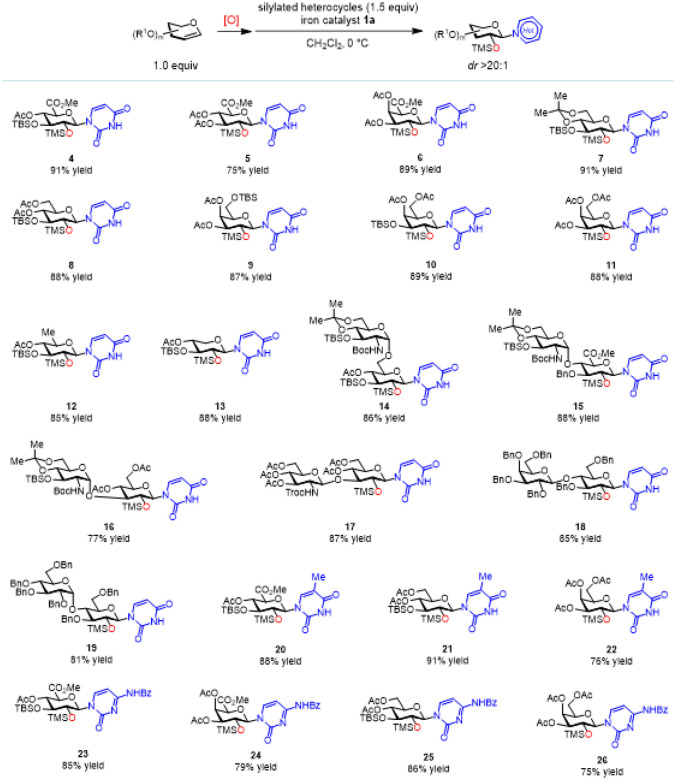
Substrate scope for the iron-catalyzed stereospecific pyrimidine *N*-glycosylation with glycal epoxides. All yields are isolated yields. See Supporting Information for experimental details.

**Figure 4:**
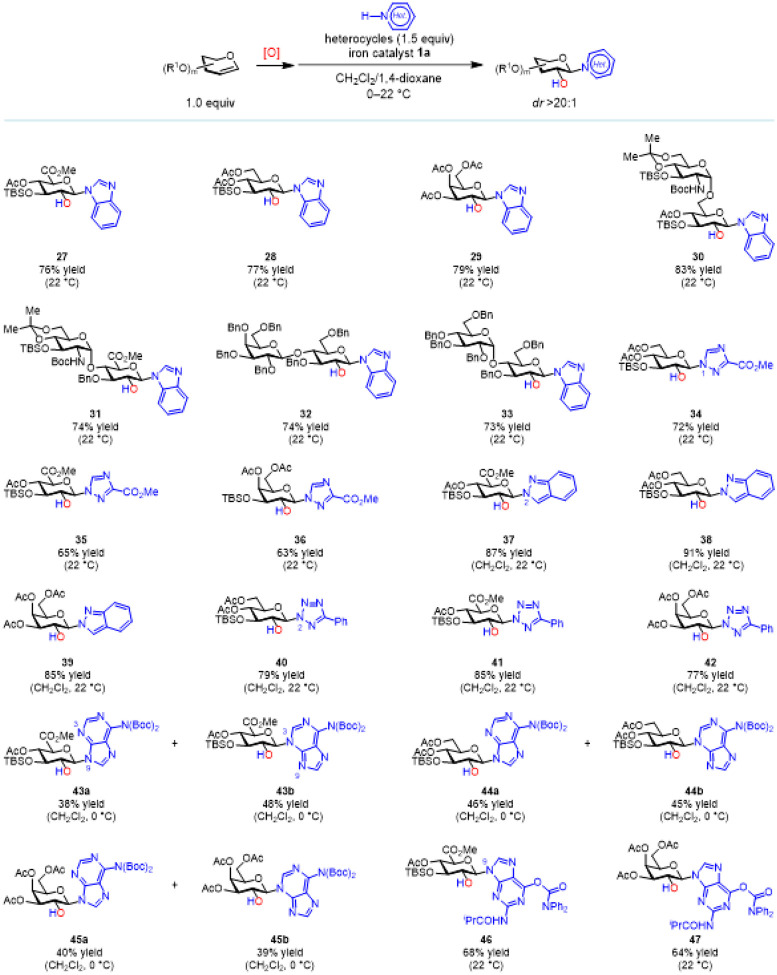
Substrate scope for the iron-catalyzed stereospecific heterocycle *N*-glycosylation with glycal epoxides. All yields are isolated yields. See Supporting Information for experimental details.
